# Water Polo Offensive Methods after the 2018 FINA Rules Update

**DOI:** 10.3390/ijerph19052568

**Published:** 2022-02-23

**Authors:** Sofia Canossa, Ricardo J. Fernandes, Luísa Estriga, J. Arturo Abraldes, Corrado Lupo, Júlio M. Garganta

**Affiliations:** 1 Centre of Research, Education, Innovation and Intervention in Sport, Faculty of Sport, University of Porto, 4200-450 Porto, Portugal; ricfer@fade.up.pt (R.J.F.); lestriga@fade.up.pt (L.E.); jgargant@fade.up.pt (J.M.G.); 2 Porto Biomechanics Laboratory, Faculty of Sport, University of Porto, 4200-450 Porto, Portugal; 3 Research Group MS&SPORT, Faculty of Sports Sciences, University of Murcia, 30720 Murcia, Spain; abraldes@um.es; 4 Neuro Muscular Function Research Group, Department of Medical Sciences, School of Exercise and Sport Sciences, University of Turin, 10143 Turin, Italy; corrado.lupo@unito.it

**Keywords:** match analysis, elite teams, water polo rules, water polo models

## Abstract

Water polo is a team sport that has been suffering rule changes aiming for a more attractive game. Our goal was to unveil whether different offensive playing styles or methods were adopted by elite national teams from Eastern Europe and from other world countries after the new rules framework was applied at the 2019 FINA World Championship. Additionally, we questioned whether those rules induced a more dynamic game. A total of 648 offensive sequences from games contested by the top-six ranked national squads were analysed. Descriptive statistics, parametric and nonparametric tests were computed, and the effect size was used. The eastern Europeans were the tallest (*t* (76) = −4.081; *p* < 0.001, *d* = 0.42) and the Hungarians were higher than Italians (*p* = 0.005, *d_z_* = −0.41). Offensive time length differed between teams (*H* (5) = 30.50, *p* < 0.001) with Serbia being the fastest (*Mdn* = 22 s). In successful attacks without extra time, Italy was quicker than Spain (17.5 vs. 25.0 s; *p* = 0.031, *d_z_* = −0.36) scoring 30% of their total goals under 20 s, while Australia up to 24% and Croatia, Hungary and Spain ≤ 16.0%. When power-play occurred, the teams’ pass action was different (*H* (5) = 15.99, *p* < 0.007), with Italy performing more passes than counterparts, especially Serbia (*Mdn* = 13 vs. 9, respectively; *p*= 0.003, *d_z_* = 0.20) and with the exception of Hungary. Through fast play sequences, Italy, Serbia and Australia scored up to 33% of their goals, while Spain, Croatia, and Hungary scored ≤ 15%. The power-play contributed to ≥50% of teams’ goals, except for Spain and Australia (48 and 45%, respectively). Playing styles commonly attributed to Eastern vs. non-Eastern Europeans and other worldwide national teams such as Australia were not confirmed. However, offensive trends were perceived and described for the first time, and some base guidelines were suggested to distinguish the static or positional vs. a more dynamic playing model. Rule changes did not seem to induce the expected effects on game dynamics.

## 1. Introduction

Water polo is one of the pioneer team sports at the Olympic Games but is progressively losing popularity. It is still considered a sport in which physical size and brute force are determinants [1,2], with sport experts questioning the game’s evolution particularly after the update of the Fédération Internationale de Natation (FINA) rules aiming for faster and more spectacular game play. This was necessary because water polo has become a mostly vertical zone style game, especially in the frontcourt attack [3,4] and it was shown that most of the players’ motions (69%) occur in a vertical body position, which is one of the reasons to consider that water polo has relatively few actions compared with other team sports [5,6].

As water polo is an invasion team sport, tactical features are one of the most important performance determinants. The playing roles are according to players’ physical characteristics and the playing methods are chosen to enhance a team’s tactical performance [7]. Until 2012, water polo game methods were dominated by the positional attack (with a lot of body contact), with the existence of a few offensive tactical combinations and driving-in movements towards the opponent’s goal to break up their defence set up (especially in an even play situation) [2]. Players’ physical features are very important given the relevance of the strength component of the game [6,8]; but, over time, the water polo community has been differentiating the teams that play using more strength movements with players in a vertical position versus those that include more swimming actions (positional or static vs. dynamic playing styles, respectively) [5]. These tactical playing behaviours (also known as “styles”) go back to the early teams, but they still meet current trends regarding how teams are playing the game [2,4,9].

Specialists stated that body strength and the ability to move quickly in the water, within the playing space, contribute to differentiating the teams’ performance and favouring unbalanced matches in female and male international championships [10,11]. In addition, it has been speculated that Eastern European squads emphasise a strength-related performance (due to players’ physical dimensions) and adopt a tactical center-dominated approach, with little frontcourt movements [1,12]. In contrast, other national teams integrating smaller body players should insist on playing a faster game to create offensive flow [13]. However, these small-bodied teams keep following the same Eastern European game approach and continue to face many difficulties when confronting taller and heavier opponents [1,13,14,15,16].

The FINA water polo rules changed in 2013 in trying to promote the flow and speed of the game, to instigate more dynamism, creativity, and velocity [9,17,18], but these were not sufficient to produce significant game play modifications. The community persists in calling for a more dynamic game with more drive-in actions and counterattacks [13,15,16,17,18,19]. Thus, at the 2018 FINA world water polo conference, rules update proposals were discussed and approved, being applied at the 18th FINA World Championships (Gwangju 2019), which could possibly change teams’ tactical behaviour and playing methods. However, no research has focused on identifying and distinguishing the static and dynamic water polo playing styles under the new rules framework. Given so, this research is the first study focused on this issue that has never been explored and is the first attempt that seeks to create a starting point and encourage broader studies in the future.

As a result of the above-mentioned, this study aims to unveil whether different water polo offensive playing styles or methods exist and to characterise them among national teams from Eastern European and from non-Eastern European and other world countries such as Australia. Furthermore, it intends to propose some base guidelines as to the classification of the eventual different offensive playing method trends such as elite offensive game models. Hypothetically, teams are still trying to follow an Eastern European game model, regardless of being constituted by lighter and shorter players. Nevertheless, it might be expected that offensive plays of those teams seek drive-in actions towards the goal in a higher percentage than those Eastern European elite teams. According to and taking into account the most recent findings about the influence of changes of the rules in the game [6,20,21], and to question whether the new regulation induces a more dynamic and fast game, special attention was given to the following rules changes: (1) reduction to 20 s of the time added for more offensive opportunities after an exclusion foul or shot (2) free throw-line shift from 5 to 6 m and (3) reduction in the time-outs allowed from four to two. Hypothetically, rule changes were not enough to induce a more dynamic game. 

## 2. Material and Methods

### 2.1. Participants

The sample comprised 648 offensive sequences obtained from 13 games played by the top-six ranked male national teams of the 18th FINA World Championship (from the preliminary round and crossover matches to semi-finals and 3rd/4th final match). A preliminary online survey was conducted to capture 11 expert opinions (international water polo coaches and researchers) about the 16 candidate teams under consideration for the world championship and their game characteristics. They were asked to point out which teams they believe typically use a more static or a dynamic game style. By crossing the outcomes of the above-mentioned questionnaire and the final world tournament ranking, it was possible to choose top national squads previously categorised from Eastern European and other world countries in an equal number. Thus, the team groups were classified as Eastern European (composed of Serbia, Croatia, and Hungary) and as non-Eastern (composed by Italy, Spain and Australia).

Matches were chosen from balanced final resulted games, i.e., their result did not surpass differences of three goals [10,15,22], and in which national teams under study would face each other. Team players consisted of all field tactical roles and no individual informed consent was requested as the event was in the public domain, with matches being broadcast by FINA tv™ and teams’ information (including players’ personal information) obtainable from official tournament OMEGA™ data. The study was conducted by respecting the Declaration of Helsinki principles and was approved by the local University Ethics Committee (CEFADE 26.2013).

### 2.2. Study Design

**Figure 1:**
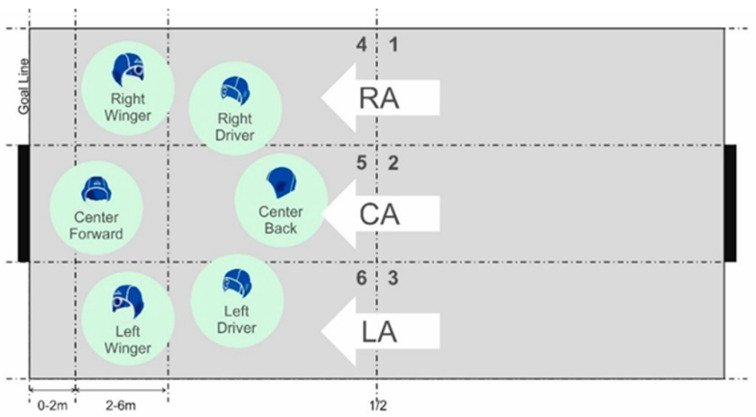
Offensive water polo field of play according to the offensive referential and playing positions; the aisles or pathways are pointed out as right, central and left (RA, CA and LA, respectively); the first receiving pass areas are defined from the right to the left as 1 to 3 and 4 to 6, before or after the midfield line, respectively.

Notational analysis was performed by one expert (the first author) watching FINA tv™ matches and using specific tactical-technical variables. Tournament scores and official game reports (start list, play by play and results) were also considered to check data, if necessary, with the intra-observer reliability achieving Kappa Index ≥ 0.91. A field of play scheme, adapted from previous studies [9,11,23], was used as an observational support instrument (Figure 1).

The observational tactical-technical variables were previously selected based on the literature e.g., [10,11,12,15], as described in Table 1.

### 2.3. Statistical Analysis

The offensive game ratios as to shots/goals and absolute and relative efficacies were calculated. Furthermore, descriptive statistics were performed (mean, standard deviation, frequencies of occurrence, median, minimum, and maximum values) and the normality of the sample distributions was checked (Kolmogorov–Smirnov). Parametric and nonparametric tests were computed accordingly to data normality and amount of cases. Thus, independent *t*-test or Kruskal–Wallis with multiple comparisons (with Bonferroni correction) were calculated. Statistics were performed with a significance level of *p* < 0.05. To estimate the effect sizes, Cohen’s *d* was calculated for the independent t-tests and Cohen’s *d_z_* for the Kruskal–Wallis and interpreted as follows [24]: small (0.10–0.29), medium (0.30–0.49) and large effect (>0.50). The statistical analyses were conducted using IBM SPSS (version 26, Armonk, NY, USA).

## 3. Results

Team basic features such as age, height, and weight and their comparison are shown in Table 2. The Eastern European teams were composed of taller players than the non-eastern group (*t* (76) = −4.081; *p* < 0.001, *d* = 0.42). Comparing teams, players’ height was dissimilar (*H* (5) = 17.24, *p* = 0.004), revealing that Hungarians were taller than Italians (*p* = 0.005, *d_z_* = −0.41). 

Between groups, the total number of offensive sequences, their time length, number of passes, ball changes of aisle through pass or number of players directly involved did not differ. In contrast, the time length of offensive sequences was different between teams (*H* (5) = 30.50, *p* < 0.001), with Serbia registering the lowest median values compared with all counterparts, especially with Hungary (22 vs. 28 s; *p* < 0.001, *d_z_* = 0.17) accomplishing 17% of their total offensive sequences until 10 s of ball possession, against 3% of Hungary. Furthermore, 63% of the offensive sequences occurred without time added according to the rules and in those attacks the offensive time length, amount of passes, aisle variation and number of players involved, occurring as described in Table 3.

When goals were scored in those sequences, the median teams’ offensive time length differs (*H* (5) = 11.57, *p* = 0.041), with Italy being faster than Spain to conclude the attack (17.5 vs. 25.0 s; *p* = 0.031, *d_z_* = −0.36) scoring 30% of their total goals under 20 s, while Australia up to 24% and Croatia, Hungary and Spain ≤ 16.0%. When the attack was extended (37% of the total sample), the teams’ median offensive time length also differed (*H* (5) = 12.93, *p* < 0.0241). In those attacks, Serbia was faster than Italy (29.0 vs. 37.5 s, respectively; *p =* 0.014, *d_z_* = 0.23), whose offensive time length was the highest of all teams in those extended sequences. 

Additionally, the number of passes per offense was dissimilar among teams (*H* (5) = 14.94, *p* < 0.011), with Serbia performing a lower median number of passes than Italy (four vs. six, respectively; *p* < 0.012, *d_z_* = 0.13). That dissimilarity between teams was also seen in offensive sequences without time added according to the rules (*H* (5) = 12.36, *p* < 0.031), with Serbia showing the same tendency for lower values. The same occurred as to power-play (*H* (5) = 15.99 *p* < 0.007), revealing that Italy performed more passes than counterparts (except Hungary), which was relevant when compared with Serbia and Australia (13 vs. 9 and 10 passes, respectively; *p* = 0.003 and *p* = 0.045; *d_z_* = 0.26 and *d_z_* = 0.20). 

Moreover, teams performed differently to aisle variations when they scored goals in attacks that did not benefit from extra time ( *H* (5) = 15.16, *p* < 0.010), with Italy executing half of the aisle variations as Spain (two vs. four variations; *p* = 0.006, *d_z_* = −0.42). That performance dissimilarity also occurred in sequences with attack time added according to the rules (*H* (5) = 12.86, *p* < 0.025), in which Serbia had fewer variations than Hungary (six vs. eight variations; *p* = 0.039, *d_z_* = 0.19). Furthermore, Serbian attacks involved up to two players in ≤35%, while their counterparts in ≤25%. Up to 48% of the total goals scored by Australia, Serbia and Italy directly involved three attackers, while their counterparts’ percentage decreased to 28%.

Both groups regained ball possession more frequently in the midfield area (when play restarted after goal) followed by the 2 and 6 m central area. The more frequent situations of repossession were after goals, followed by the goalkeeper’s defense, ball steals in the opponent center forward, offensive fouls, and defensive blocks. Furthermore, the Eastern European group tended to perform the first pass to previous and adjacent midfield line areas less frequently than non-Eastern ones, mainly to the right aisle (14 vs. 22%). By teams, ball possession was regained similarly as to what was observed in groups, but Serbia tended to regain it more frequently through defensive stealing in the opposing center forward (23%) than other actions, which contrasted with Hungary’s defensive steals (11%). In addition, the Serbian team also frequently performed the first pass towards the left aisle, in contrast to counterparts. Moreover, although there is a similarity between groups regarding initial sprints, Italy, Spain and Australia tended to win them more than Eastern European teams (83, 58 and 92 vs. ≤ 33%, respectively). 

**Figure 2:**
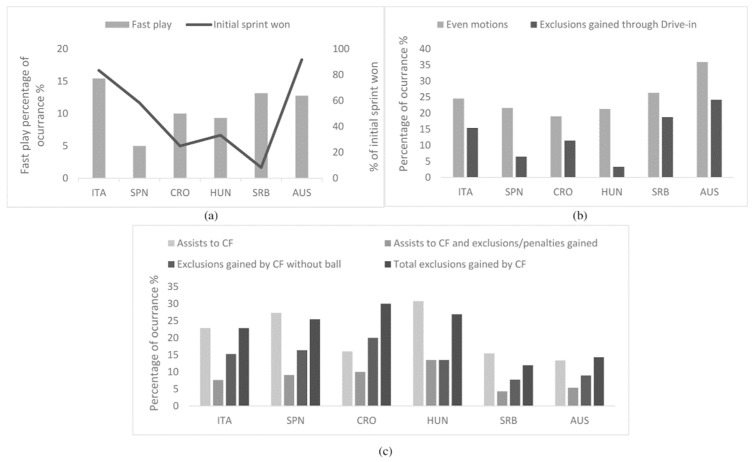
Teams’ main offensive methods and behaviours up to and during the positional even play: (**a**) fast plays and initial sprints won; (**b**) actions in even play and consequent drive-in exclusions gained; (**c**) assists to center forward and exclusions gained with or without ball. CF – center forward player; ITA—Italy; SPN—Spain; CRO—Croatia; HUN—Hungary; SRB—Serbia; AUS—Australia.

In the transition phase, the most frequent defensive behaviour by groups and teams was the non-pressuring opposition. The Eastern European group tended to face the active opposition more often than the counterpart group (36 vs. 27%) and, in even play, following the mix-floating (more frequent), they tended to meet less individual defense than the non-Eastern group (9 vs. 13%). By teams, Serbia and Australia were those who faced more individual, close pressuring defenses and defensive system variations during the same offensive sequence. Concerning offensive methods, both groups performed the positional attack (≥80%) more frequently than the fast attack and counterattack. Furthermore, in even play, ball circulation and the joint movement of players, with the body in a vertical position during a center forward duel, was prevalent as teams’ tactical behaviour in ≥74%. The initial sprints won, fast play methods, tactical behaviours in even play, and the subsequently earned exclusion fouls are shown in Figure 2.

Through fast play, Italy, Serbia and Australia achieved 33, 26 and 24% of their total goals, while Spain, Croatia and Hungary achieved ≤15%. Furthermore, regardless of subsequent offensive actions, 38% of Australian goals came from attacks in which drive-ins occurred, and in Serbia, Spain and Italy, that happened in ≥29% of goals, contrasting with Hungary and Croatia in ≤18% of goals. Likewise, in offensive sequences, in which center forward was assisted, ≥21% of Spanish, Hungarian and Croatian goals emerged in contrast with ≤13% of goals from Serbia, Italy and Australia. Of all center forwards’ involvement during the offensive process and all exclusions of its center defender earned, Hungary and Croatia attained ≥ 48% of their goals, Spain and Italy ≥ 41% and Australia and Serbia ≤ 31%. 

Both groups had a similar proportion of offensive sequences in which time was added according to the rules, as well as the number of situations that led to those extensions. However, between teams, while Croatia and Hungary had 41% of their total sequences benefiting from offensive time added, Serbia and Australia had ≤34%, who performed the most offensive sequences without any extended time. The exclusion foul (and subsequent power-play situation) was the main reason to obtain more offensive time, which occurred in 26–40% of matches (min-max sample values registered in Serbia and Hungary, respectively). Hungary tended toward the highest power-play efficacy values and Spain the lowest (49 vs. 39%, respectively), but that situation contributed to more than half of the teams’ total goals except in Spain and Australia (48 and 45%, respectively).

The power-play was mostly performed through a 4:2 rotating tactic (up to 69% listed in Spain), but Italy preferred to perform the fixed 4:2, although it tended to be more effective in changing tactics (67%). Australia and Croatia tried more often to perform the quick play, contrasting with the remaining teams (22 and 18% vs. ≤13%, respectively), being 63 and 57% effective. Additionally, Serbia tried to score through the 6 m free shot more often (11% of occurrence) than counterparts (≤7%), and, regarding the time-out strategy, Hungary and Australia showed the best efficacious results (60 and 75%, respectively). Serbia and Italy scored 33 and 25% of those situations, Spain and Croatia did not score. 

## 4. Discussion

Obtained results are in accordance with the author [1], who reported that Eastern European water polo players are the tallest in the world. Furthermore, as previously reported, the general physical characteristics of all players highlight that water polo players are tall and have a large body mass [25]. It is known that a better body composition of highly competitive level players has a strong correlation with conditioning and performance factors [26] and, although no differences were detected in the present players’ body weight, maximum values reached 130 kg. Results confirm that players must be tall and strong to face the required game tasks, such as passing, jumping, blocking, and defending [1,27]. However, Italian players (world champions) were shorter and tended to be the lightest of the entire sample, highlighting the importance of looking at tactical factors, teams’ playing procedures and their main choices as opposed to offensive methods [11].

According to Gardasevic et al. [21], with the rule changes in reducing the 2nd attack to 20 s, the game started to present more ball possessions. For the authors, this had an impact on the total number of attacks, faster swimming, and more frequent shots, which appear to be associated with the winning teams. However, the present number of offensive sequences was not different between groups and teams, and the total ball possessions seemed to be lower than those previously reported regarding the winning teams [23]. This can be due to different study methodologies since the number of possessions is impacted by the number of exclusion fouls and other events that increase offensive time and add to the length of a possession [28]. This is in agreement with the present study since it considered that ball possession encompasses all the offensive events that happen up to total ball loss and the team’s defensive recovery taking place. The exclusions fouls do not settle the end of ball possession, and, in relation to Canossa et al. [23], it seems that currently more exclusions fouls occur (27 vs. 33%, respectively).

Serbia’s less time spent per offensive sequence, benefiting or not from offensive time extension according to the rules, seems to contradict researchers who found that winners were those who use less time in ball possession e.g., [29]. In fact, Serbia was the 5th in the final ranking and the winner (Italy) presented the highest possession length regarding the total sequence’s mean values. Although Italy registered lower time length in attacks that did not benefit from time extension, it was also the team that spent the longest time concluding sequences with power-play, while Serbia spent the least. Moreover, regardless of Italian and Serbian teams’ similar frequencies of fast play (counterattack and fast attack), Italy’s success agrees with the literature that reported that winners are the most effective in counterattacks [11,29]. Furthermore, Saavedra et al. [20] found that winners’ efficacy was greater (12%) after the rule changes, which agrees with present results.

Concerning the number of passes performed, a previous study reported an average of four passes in even situations, while, in power-play, the number almost doubled, concluding that possessions with fewer passes were more effective [30]. Likewise, more recently, a mean of four passes per offense was found [9]. Current values disagree with those reported, except for Serbia; however, when the offensive time was not extended, Italy and Australia were effective with fewer passes (three, respectively), while, in the extended sequences (mostly with power-play), Italy performed more passes (13, respectively). Similarly, Serbia increased its number of passes in extended sequences, which is in line with Hughes et al. [30]. The divergencies in study methodologies could contribute to the differences found in the present values to in those reported in the literature; however, it can also be due to rule changes and evolution of power-play performance, which is considered a key factor for game success [28].

Concerning aisle variations, the results may indicate the teams’ procedure preferences in quickly trying to send the ball deep into the playing space, directly to the opponent’s target, or, passing wide, attempting to create shooting opportunities (with more aisle variations). Teams can combine playing methods throughout the game or in the same offensive sequence, as observed in other sport games [31,32,33]. Present results suggest that Italy can be one of those cases since it tried more fast plays using fewer aisle variations but, in attacks with extra time, performed distinctively from Serbia, with a much higher number of aisle variations. Furthermore, the number of players directly involved was similar to those found by Lupo et al. [22], corroborating those authors’ conclusion that, in power-play, better teams involve more players. Thus, current results agree with the statement that power-play is clearly advantageous to score, tending to have an elaborate preparation, unless the quick tactic is used [15].

Regarding the means of how the ball is repossessed, besides the goalkeeper’s defense, whose performance is widely reported as a success indicator e.g., [20,29], the defensive stealing was also currently observed, such as in earlier game analysis studies [29,30,34]. This action was recently considered a success indicator [20] and is important evidence of a team’s intention to counterattack, as was observed in Serbia. This latest team also tended to progress in the field by performing the first pass to the left aisle more often than its counterparts, suggesting the intention of assisting their left-handed teammates nearer the opposite goal, which can be due to its apparently higher shot efficacy than right-handed players [35]. Another important conduct of regaining the ball was winning the initial game sprints, which was considered discriminatory of winners from losers [20] and, in the present results, the 1st and the 2nd classified teams won the initial sprints more than the Eastern Europeans.

Additionally, non-Eastern teams’ tendency towards an active initial defensive pressure over the Eastern Europeans may indicate the demand to first (immediately) stop the opponents’ advance and then quickly return to cover the central pathway to organise their defense. Their defensive arrangements will aim to cover the center forward zone [11,12] and protect the goal with defensive blocks, avoiding physical contact with Eastern European players considered physically powerful [1]. This suggests that Italy, Spain and Australia tend to exert more initial pressure and be more vigorous and agile than Eastern Europeans. On the other hand, having large physical dimensions, the Eastern Europeans do not need to oppose by using physical contact, as their space occupation and defensive blocks help a lot to protect their goal. However, Australia and Serbia tend to face more individual defense and system variations from their opponents, which seems contradictory. However, a certain opposition can be the result of the opponents’ scouting and their technical decisions [36] of constraining drive-in actions, causing failed passes and hasty shots when facing teams such as Australia and Serbia.

The present results lead to infer that Eastern European and non-Eastern groups do not correspond to a possible static vs. dynamic style of play, respectively. The distribution of fast plays, drive-in actions, and the total involvement of the center forward in the offense looks very heterogeneous, specifically highlighting that Serbia’s team seems to not have been well grouped. According to recent research with the new rules framework, counterattack shots, 6 m free throws and center forward shots decreased. Furthermore, those rules led to a greater number of attacks and shots in total, more power-plays and goals scored, being stated that the game became more dynamic and faster [20,21,37]. However, the current results lead to induce that, although the game seems more dynamic, with more power-plays, more attacks, and goals, this can mean more time in front of the opponent’s goal (since one offensive sequence can have more than one time extension and shot opportunity) and more game interruptions to replace the ball in the midfield and restart the match.

Following the previous idea as to the effects of the rule changes, whether power-play and goals had increased, then, teams’ offensive behaviour can be in accordance with the ones stated by Graham and Mayberry [15] that, in balanced games, teams are obtaining their goals in the same way. In fact, as the authors state, although some teams try to perform more drive-ins than others, the outcome is similar, i.e., obtaining exclusion fouls to benefit from power-play situations. In their research, it was found that the exclusion conversion rate was the most effective discriminatory statistic (correctly classifying almost 90% of all contests) and, on average, winners scored 18% more power-play situations than losers. The present min-max results appear less divergent (10%) than those reported by Graham and Mayberry [15], but teams power-play goals in their total goals (45–68%) seems to be discrepant. This can be due to Serbia’s minor trend in achieving power-plays, yet their efficacy surpassed Italy and Croatia, with Spain being the less successful. With the new rules and the reduction in the time available to perform power-plays, it seems that the ability to score in that situation in the game can be even more crucial [38].

Thus, in the present rules framework, quick power-play tactics should be more frequently played, not only due to time reduction to solve the power-play situation but also because quick tactics are of great efficacy according to the literature [15,38]. Italy seemed to be more effective as to quick power-play tactics, but they undertook more frequently a fixed and open 4:2 system with ball passes from winger to winger (crossing the three corridors), creating space and trying to obtain an open shot angle or assist 2 m players. Most probably, Italy’s power-play tactical choice was linked to its players’ general physical characteristics, lesser height and its high level of passing skills. However, debating the rule changes with regard to the effect on power-plays [37], their higher frequency of occurrence reinforces the idea that players now spend more time in front of the goal in a positional attack with the body in an upright position. This, adding to the greater amount of even play in the game [26], performed mainly in a vertical position, suggests that rule changes did not induce very positive effects in the matches, which is in line with previous research [6].

The increase in penalties was another effect of the rule changes reported [21], but, in the current research, they mainly occurred in Croatia’s team. However, results are still in agreement with a study that reported that penalties and power-plays together account for about 56% of goals in elite matches [39], meaning that more than half of the goals come from disciplinary sanctions for serious fouls. Thus, it seems that body contact or wrestling bouts between opponents within the game [8,22,26] still remain at a high rate. In addition, the reduction in counterattacks was also reported as a consequence of rule changes [21,37], which may have a negative effect not only in game content but also on its spectacularism. The fast break is one of the most exciting game moments although less frequent [4,13] and, in the present study, remained the most effective playing method, in agreement with the literature [20,22].

Additionally, recent studies found that the change of the direct free-throw line to 6 m away from the goal affected that type of shot frequency [20,21], which was also perceived in the current results, although Serbia tried it more and was efficient. Since in previous research [15] the free throw was the most efficient strategy in even play, the change of its distance does not seem to produce a positive effect. This view is corroborated by the fact that even play is the most frequent situation in the game, and it was found that it has a greater probability of not scoring a goal [23]. Perhaps game progress will go through the revision of tactical actions and playing methods for even play success and the center forward role settled in the 2 m line in front of the goal [4,6,14].

Following the effects of the new rules, the decrease in time-outs reduced the players resting time during the match but also the coaches’ intervention in trying to improve team performance, mainly in power-play. The present results are in line with the recent research that found that some coaches mistakenly believe that time-out helps to reach more power-play success [38]. However, in balanced games and elite competitions, time-outs can reflect the value of the coach’s decision-making at specific moments of a match [20,38]. In the current championship, Hungary won the quarter-final game in the last 3 seconds of the match, after a time out, which took Australia out of the semi-finals. Thus, like some authors [10] have clarified, in balanced games, it becomes difficult to point out technical-tactical aspects that can distinguish teams, since their differences will be reduced with little significant effect. Thus, it would be important to consider the game as a complex and dynamic system pondering other variables to explain the results [40].

This study suggests that Serbian performance was quite dissimilar from Eastern European counterparts. It is possible that their team composition with slightly younger players helps to explain it; however, their tactical options seemed to be more in line with an eventually more dynamic game model. Contrarily, Spain appeared to exhibit offensive procedures leaning toward an eventually more static or positional model, revealing a trend toward a center forward approach [12]. Italy seemed to reveal both offensive procedures since it won several initial sprints, undertook fast play sequences with efficiency, had the lowest times spent in non-extended offensive sequences and performed drive-in actions in even situations. Furthermore, Italy had the shortest and lightest players of the studied teams. However, it also exhibited a greater center forward involvement in even plays and had the highest time spent in the extended sequences in the positional attack.

This study’s main hypothesis as to the correspondence of groups to given playing methods and behaviours was not confirmed; however, although Italy was revealed to be pursuing a more dynamic style of play, they are still possibly trying to follow a static game model. Furthermore, Spain showed a tendency toward a more static or positional play and a center forward approach, implementing fewer fast play methods. Thus, we would group Spain, Croatia and Hungary into a dominant positional playing model and Italy, Serbia and Australia into a wiggling, dynamic playing model. As to rule changes, despite the authority’s good intentions and efforts regarding water polo improvement, recent rule changes do not seem to promote a more dynamic and spectacular game, fostering more tactical and intelligent behaviour. Moreover, the wrestling game characteristic prevails as well as the great dependence of the center forward position and its influence on offensive actions and team choices [6]. As some experts consider, the center forward role seems to be conditioning the game evolution [14,18].

Water polo game models have never been made on successive rule changes, and their knowledge can be crucial not only for coaches to consider game options according to team characteristics and guiding their activity but also for water polo authorities to perceive game evolution tendencies and be able to properly implement rule changes that favour it [9,16]. 

Taking into account the present results, other contributions and literature [2,6,15,38], base guidelines to identify the dominant positional playing model are suggested: (1) teams with players that are taller, heavier, and have a great wingspan (average body height ≥ 193 cm); (2) performing few fast play sequences in the game (less than 15%); (3) performing less than 24% of the total amount of sequences up to 25 s; (4) exhibiting offensive sequences with ≥25 s of median time, seven passes and five aisle variations or more; (5) performing the even play mostly through the positional attack method with ball circulation and a center forward approach (few drive-in motions); (6) exhibiting more than 35% of extended sequences in which power-play is prevalent; (7) the power-play is performed with more than eleven passes and quick tactics are infrequently used.

Additionally, as base guidelines to identify the offensive wiggling, dynamic playing model, the opposite of the above-mentioned is suggested. Still, we admit the existence of other playing models that would be useful to know and explore. The present study had the limitation of being developed with a small sample and only one championship, and it is now suggested that further research should be carried out in different championships, playing levels and genders. It would be valuable to have more information that would provide the understanding of the prevalence or not of game models and the revision of guidelines for their appreciation.

## 5. Conclusions

The current study represents a first attempt to verify and suggest some base guidelines to distinguish what is consensually and empirically accepted by the community as being static vs. dynamic game styles in the new rules framework. The main study hypothesis was not confirmed since it was not possible to establish the correspondence of an eventual static vs. dynamic playing style of Eastern European vs. non-Eastern teams as they were initially grouped, meaning that the consensual playing methods commonly known by the community do not currently correspond to specific countries. Serbia seemed to exhibit faster and more dynamic playing procedures than all the other teams and Italy presented dynamic tactical methods but also dominant positional playing procedures revealing the longest offensive sequences when the time was extended by the rules. Thus, given Italy’s team characteristics and its players, it is possible that they are still following a static game model, as it historically has been imposed by greater elite Eastern European teams, mainly regarding center forward play and elaborated power-play. 

Since national team groups previously established for this study did not meet with the hypothesised offensive methods, it was suggested that Spain, Croatia, and Hungary should be considered as one group that performed a basically positional playing model, and Italy, Serbia and Australia as another group that presented a more offensive wiggling playing model. Furthermore, since water polo game models were never featured in this century and throughout all rule changes, this current study suggests, in a first attempt, some base guidelines to identify those playing models. Regarding rule changes, although authorities had good intentions to enhance the game, those changes do not seem to promote a more dynamic and spectacular game. Water polo requires a greater investment in studies that support eventual decisions regarding regulatory changes to effectively impact game evolution.

## Figures and Tables

**Table 1:** Tactical-technical variables considered for the offensive sequences analysis.

Variables	Description
Offensive sequences (*n*)	Team possessions per game, with or without time extension (since ball possession until total ball loss to the opposing team, with or without time added by exclusion foul, corner or rebound).
Offensive time (*s*)	Offensive sequence duration (total clock time spent in the offensive sequence).
Recovery area (*n*)	Field of play area where theball was recovered (including the restarting play).
Ball recovery (*n*)	Method of how the ball was gained or repossessed (i.e., initial sprint won, goalkeeper defence, failed shot, defensive block or rebound, goal suffered, opponent center forward foul, ball steals, bad pass, opponent time expired, game periods or match over).
First aisle or pathway (*n*)	Field area and aisle of the first pass (dribbling was also considered).
Defensive opposition (*n*)	First opponent defensive methods at the beginning of the offensive sequence (passive, active or without setup by expired time, timeout or game over).
Defensive setup facing positional attack (*n*)	Individual (man to man), total zone (or cluster), mix-floating (zones with blocks combined and “w” defence) or changing the initial defensive setup (from individual to mix and vice versa).
Total passes (*n*)	Occurred in each offensive sequence.
Ball aisles variations (*n*)	Occurred through passing action.
Field players involved (*n*)	Handling the ball within each offensive sequence.
Team offensive methods (*n*)	Fast plays (counterattack and fast attack) and positional attack (even-play and power-play).
Tactical even behaviours/actions (*n*)	Drive-ins to build up score situations (outwards and inwards to reach center forward position, pick and screen and switching), ball circulation in a vertical position and center forward duel (with and without ball).
Individual tactical means(*n*)	Drive, bounce, fake shot, 6 m free throw or center-forward shot.
Power-play methods and systems (*n*)	Quick, 4:2 or 3:3 tactical setup (with or without changes as to initial form) or 4:2 rotating.
Sequence outcome (*n*)	Aborted (without shot occurrence), unsuccessful (shot went out of the field), partial success (ball hit goal post/crossbar, or was defended by the goalkeeper), or success (goal).

**Table 2:** Mean plus standard deviation (M ± SD), minimum-maximum (min-max) and median (Mdn) values of each team’s basic features and their comparison.

Groups	National Teams	Age (Years)	Min-Max	Mdn	Height (cm)	Min- Max	Mdn	Weight (kg)	Min-Max	Mdn
Eastern European	Croatia	28.9 ± 4.0	22–35	28	194.6 ± 1.6	185–203	196	100.4 ± 12.6	84–130	96
	Hungary	26.8 ± 3.4	22–34	27	197.4 ± 1.2	192–203	197 *	96.6 ± 8.5	82–108	98
	Serbia	24.7 ± 2.3	21–29	25	195.2 ± 1.1	190–202	195	95.3 ± 3.9	91–101	94
Non-Eastern European	Italy	27.7 ± 4.2	21–35	29	189.6 ± 1.4	180–198	190 *	91.2 ± 8.8	76–104	92
	Spain	26.7 ± 5.1	20–38	25	191.2 ± 1.7	181–203	191	94.2 ± 9.2	84–110	90
	Austrália	26.8 ± 3.7	20–32	27	192.5 ± 1.4	186–200	193	101.4 ± 12.1	87–130	98

* Significant differences among teams (*p* = 0.005).

**Table 3:** Teams offensive sequences without time added according to the rules and their description (mean ± SD) of the quantitative tactical-technical variables considered.

Teams	Sequences without Extension (%)	Time Length (*s*)	Total Passes (*n*)	Aisle Variations (*n*)	Players Involved (*n*)
Italy	61.9	21.0 ± 7.1	4.2 ± 2.4	2.7 ± 1.5	3.3 ± 1.2
Spain	62.7	23.5 ± 7.2	4.4 ± 2.5	3.2 ± 2.0	3.3 ± 1.3
Croatia	59.0	22.2 ± 7.0	4.1 ± 2.1	3.2 ± 1.7	3.0 ± 1.1
Hungary	58.6	22.3 ± 6.7	3.8 ± 1.9	2.7 ± 1.4	3.0 ± 1.1
Serbia	65.8	17.5 ± 8.6	3.4 ± 2.3	2.5 ± 1.9	2.9 ± 1.5
Australia	66.1	21.9 ± 6.6	4.1 ± 2.4	3.2 ± 1.7	3.2 ± 1.1

## Data Availability

Raw data of this article are available upon request to corresponding author.
